# Fabrication of Through via Holes in Ultra-Thin Fused Silica Wafers for Microwave and Millimeter-Wave Applications

**DOI:** 10.3390/mi9030138

**Published:** 2018-03-20

**Authors:** Xiao Li, King Yuk Chan, Rodica Ramer

**Affiliations:** 1 School of Electrical Engineering and Telecommunications, University of New South Wales, Sydney, NSW 2052, Australia; kyc@unsw.edu.au (K.Y.C.); ror@unsw.edu.au (R.R.)

**Keywords:** microwave and millimeter-wave, through via hole, fused silica, DRIE, laser ablation, etch rate

## Abstract

Through via holes in fused silica are a key infrastructure element of microwave and millimeter-wave circuits and 3D integration. In this work, etching through via holes in ultra-thin fused silica wafers using deep reactive-ion etching (DRIE) and laser ablation was developed and analyzed. The experimental setup and process parameters for both methods are presented and compared. For DRIE, three types of mask materials including KMPR 1035 (Nippon Kayaku, Tokyo, Japan) photoresist, amorphous silicon and chromium—with their corresponding optimized processing recipes—were tested, aiming at etching through a 100 μm fused silica wafer. From the experiments, we concluded that using chromium as the masking material is the best choice when using DRIE. However, we found that the laser ablation method with a laser pulse fluence of 2.89 J/cm^2^ and a pulse overlap of 91% has advantages over DRIE. The laser ablation method has a simpler process complexity, while offering a fair etching result. In particular, the sidewall profile angle is measured to be 75° to the bottom surface of the wafer, which is ideal for the subsequent metallization process. As a demonstration, a two-inch wafer with 624 via holes was processed using both technologies, and the laser ablation method showed better efficiency compared to DRIE.

## 1. Introduction

Fused silica (SiO_x_) and quartz wafers are widely used in planar microwave and millimeter-wave circuits, due to their low loss properties and good thermal shock resistance. Microstrip technology has become the best known planar transmission line medium for radio frequency (RF) and microwave circuits. The popularity and widespread applications are due to microstrip line circuit’s planar nature, ease of fabrication using the various processes available, easy integration with solid-state devices, and good mechanical support. The ground plane connection plays a critical role in microstrip line circuits, as when a high frequency signal is conveyed in microstrip transmission mode, the energy is concentrated between the signal line and the ground plane. At the same time, it can affect the transition from coplanar waveguide (CPW) mode to microstrip mode. Transitions between microstrip and CPW were proposed and measured in [1,2,3], and the results showed that the ground plane implementation has a large impact on the bandwidth, insertion loss, as well as the chip size. With the increase in frequency, a thinner substrate is preferable [4] in order to reduce the dispersion and radiation loss. The microstrip line dispersion is proportionate to the ratio of substrate thickness to effective wavelength, so the substrate thickness should be decreased to maintain the required dispersion level at higher frequency [5]. Thin substrates also need a narrower microstrip line width to keep certain characteristic impedances, which allow the miniaturization of the structure and reduce radiation.

The traditional methods to make ground connection are radial stub virtual ground bonding and wire bonding. Radial stubs can reduce the costs of fabrication [6]; however, they will lead to unpredictable resonance which can be eliminated by silver paint ground connection [7]. Wire bonding is another option to make ground connections, and ribbon bonding could highly reduce the parasitic inductance compared to normal wire bonding. Despite the improvement in bonding techniques, proper capacitive compensation circuits need to be applied [8]. Besides this, dicing and bonding are deemed not suitable with pre-packaged RF micro-electromechanical system (MEMS) structures. As a result, a better ground connection method is required, which leads to the suggestion of through via holes in glass wafers.

Moreover, 3D RF integration has become popular in recent years due to the continuously increasing demand for product miniaturization, high package density and better performance. Compared to other substrates used for passive microwave components, such as polytetrafluoroethylene (PTFE) and alumina, fused silica is viewed as a prospective material and has attracted a great deal of interest in RF integrated packaging applications. This is because fused silica wafers are homogeneous materials which offer compatibility with commercial wafer processes, dimensional stability against heat and humidity, and scalability when production shifts to the panel process from the wafer process [9]. Microwave components, such as spiral inductors, filters and interconnections using fused silica wafers were simulated and analyzed in [10,11]. The return loss, the insertion loss and the isolation show that using fused silica as the interposer material provides obviously improved RF performance compared to silicon material. Also, using through via holes for antennas could enable new integration and packaging opportunities in the millimetre-wave range. In [12], a directional through via holes antenna was designed and fabricated for point-to-point wireless communications, at a centre frequency of 78 GHz.

Researchers in process engineering conducted different micromachining technologies in order to fabricate through via holes in quartz and fused silica wafers. These technologies were sorted and summarized in [13]. Chemical machining using DRIE and thermal machining using laser ablation are the two solutions which can accommodate ultra-thin wafers and offer a good profile for the subsequent metallization. In [14], using a single-layer KMPR photoresist mask, up to 30 μm deep via holes were successfully etched with an etch rate about 0.5 μm/min and a mask selectivity over 1. Yu-Hsiang Tang et al. used 5.5 h to etch through the 150 μm thick fused silica wafers with the optimized process parameters obtained from experiments [15]. It also suggested that, compared to a Ni-Co metal mask, a KMPR photoresist mask is more suitable for fused silica through via hole etching. Using a nanosecond and femtosecond laser to etch high quality, high aspect ratio and crack-free microfeatures in glass substrates were reported in [16,17], and through glass via interposers used in 3D integration were fabricated in [18]. However, etching through via holes in ultra-thin fused silica wafers using laser ablation has been less explored.

Considering the wide range of applications, fabrication processes using DRIE and laser for etching through via holes in ultra-thin fused silica wafers were studied and optimized, and are reported in this paper. The pros and cons of both methods were analyzed based on experimental results. To our knowledge, although both technologies have been explored in the literature separately, there is no literature research comparing the two technologies and analyzing their feasibility for through via holes etching in ultra-thin fused silica wafers.

## 2. Experiment

### 2.1. Inductively Coupled Plasma-Reactive Ion Etching (ICP-RIE) of Fused Silica

**Figure 1:**
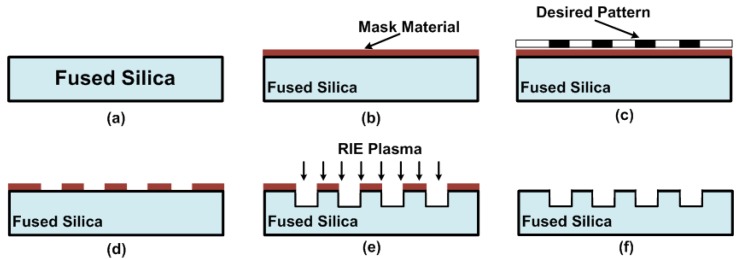
Through via hole fabrication process by deep reactive-ion etching (DRIE): (**a**) Sample cleaning; (**b**) Deposition of mask material; (**c**) Transfer of desired pattern; (**d**) Desired pattern achieved; (**e**) Inductively coupled plasma-reactive ion etching (ICP-RIE) dry etching; (**f**) Removal of mask residual.

A typical through via holes fabrication process using the inductively coupled plasma-reactive ion etching (ICP-RIE) dry etching method is shown in Figure 1. The etched double-sided polished fused silica sample had a diameter of two inches and a thickness of 100 μm.

First of all, the sample had to be properly cleaned in piranha solution, acetone and isopropyl alcohol. Secondly, a layer of mask material was deposited on the wafer and this layer will protect the wafer from plasma attack in the non-via area. Thirdly, the transfer of desired patterns to the etching mask was carried out. Then, the two-inch sample was attached to a four-inch silicon carrier wafer with aluminium cooling grease (from Amerasia International Technology, Inc., West Windsor, NJ, USA) and etched in an ICP-RIE system. Finally, the mask material was removed.

Three different types of mask material were tested and compared in the etching process, including KMPR 1035 (from Nippon Kayaku, Tokyo, Japan) photoresist, amorphous silicon (a-Si) and chromium. The KMPR 1035 was spin coated at a speed of 2000 rpm to get 70 μm film thickness. Then it was soft baked at 30 °C for 10 min, followed by 50 °C for 10 min and 100 °C for 30 min. After exposing it to 2200 mJ/cm^2^ UV light, the sample was post-baked at 100 °C for 4 min and developed in SU-8 developer for 6 min.

Unlike using photoresist alone, which can be directly patterned by photolithography, an extra photoresist mask layer is needed when transferring the desired pattern to the a-Si or chromium mask. The a-Si mask was deposited using the Oxford PECVD (Oxford Instruments, Oxfordshire, UK) with a deposition chamber pressure of 1500 mTorr, temperature of 300 °C, helium gas flow of 475 sccm, SiH_4_ gas flow of 25 sccm, and an RF power of 20 W. The desired pattern was then transferred to a-Si mask using RIE dry etching with negative photoresist nLOF2020 (from Microchemicals GmbH, Ulm, Germany) applied as an etching mask. The chromium mask layer was deposited with a HHV (Hind High Vacuum Company Private Limited, Crawley, UK) magnetron sputtering system with 600 W direct current (DC) power, an argon gas flow of 15 sccm and a deposition pressure of 15 mTorr. Similarly, nLOF2020 photoresist was used as an etching mask and chromium was etched in chromium etchant (from Sigma Aldrich, St. Louis, MO, USA).

After the deposition and patterning of the mask materials, the fused silica samples were etched in the STS ICP RIE system (Surface Technology Systems Plc., Newport, UK). According to [14,15,19], fluorocarbon plasma $CF_{x}^{+}\left(x=1,\;2,\;3\right)$ with high energy can slowly react with SiO_x_. At the same time, the combination with inert gases will boost the etch rate of fused silica by physical bombardment. Since the etch rate is very slow, this becomes the key criteria when exploring the etching recipe. By varying the process parameters and considering the available conditions in the lab, the best process characterization was found to be: 300 sccm helium flow, 30 sccm C_4_F_8_ flow, 1400 W inductively coupled plasma (ICP) power, 400 W bias power, 10 mTorr chamber pressure, and the backside cooling temperature was set to 20 °C.

### 2.2. Laser Ablation of Fused Silica

Compared to the dry etching method, using laser ablation turns out to be a simpler process for etching through via holes in fused silica wafer. It does not include mask layer deposition and the conventional photolithography required for the desired patterning. Here, 2D high precision machining is applied to draw the desired pattern. In our experiments, the machining station (Microstruct-C), with Scanlab’s galvo scanners for marking and an XY table for sample positioning, offered a positional error of less than 1.5 μm. Since the through via holes used in microwave applications were fabricated as described in the first step, alignment marks form at the same time, which are fully compatible with the following semiconductor fabrication processes.

Our laser system (Coherent Super Rapid-HE, Coherent, Inc., Santa Clara, CA, USA), used for the ablation process, can produce 10 ps pulsed laser with a repetition rate of 20 kHz, and the beam is delivered through a F = 100 mm, f-theta lens, producing a 15 μm spot at focus. To etch the very thin fused silica wafer, the second harmonic output, which gives a wavelength of 532 nm, was chosen, because this leads to more efficient non-linear absorption and smaller laser spot size. 

**Figure 2:**
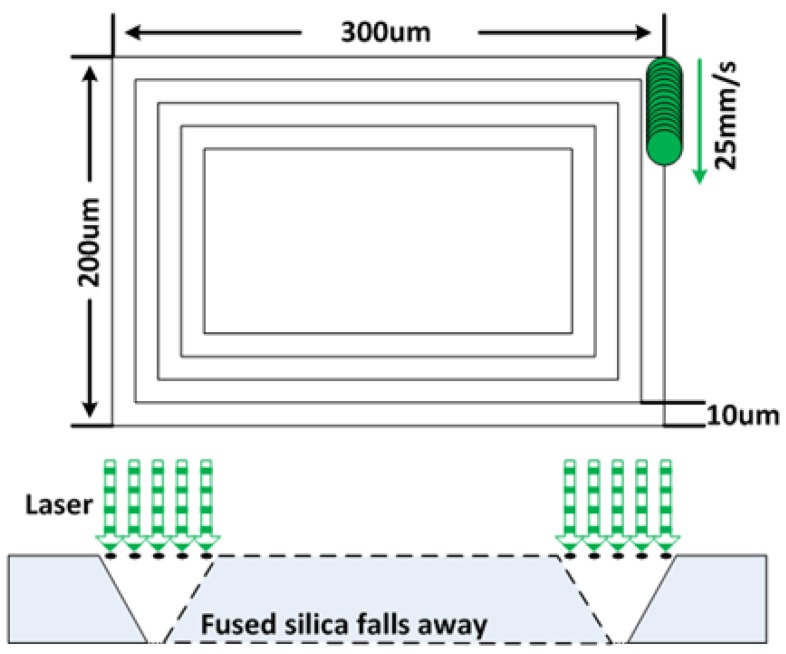
Methodology of laser ablating fused silica.

The laser ablation of fused silica will leave debris around the etched patterns, and the debris is difficult to remove without mechanical wiping or polishing. Consequently, photoresist is coated on both sides of the wafer as protection. Then, the fused silica wafer is suspended above a large silicon plate with glass slides supporting its edges. When processing the 100 μm thick fused silica wafer, we found that it was very fragile and prone to becoming chipped and even cracked when processed at high powers and speeds. Cracks on and in the wafers are problematic in microwave circuits as they will form short-circuits or introduce additional equivalent radial stubs in the subsequent metallization steps. In consequence, the etching strategy adopted was to use low power, within an order of magnitude of the glass damage threshold, and with many repetitions of the pattern in order to gently cut through the fused silica wafer. A set of five concentric squares with 10 μm offset was used as the etch pattern to create a 50 μm kerf for the centre to fall away in a ‘non-contact’ fashion, as shown in Figure 2. The scanning speed of laser beam was set to 25 mm/s in equivalent to a pulse overlap of 91% to create a smooth etching result.

## 3. Results and Analysis

### 3.1. ICP-RIE of Fused Silica

The experimental results indicate that the etch rate is mainly proportional to both ICP power and bias power, because the ICP power determines the plasma density in the chamber and the bias power gives the etching momentum of the high energy ions. We also found that the selectivity between the fused silica and the mask material decreases when the bias power is higher, so there is a trade-off when determining the bias power. The etching results obtained by the optimized process conditions are shown in Table 1.

The highest achieved etch rate was 0.52 μm/min, so it needs at least 3.5 h to fully etch through the 100 μm fused silica wafer. During the long period of continuous etching, a lot of heat is generated by ion bombardment and then accumulated, which can seriously burn the photoresist mask and lower the etching selectivity between fused silica and mask material. It should be noted that the burned photoresist is very difficult to remove. This burning cannot be avoided even though different measures have been taken. The sample was attached to a four-inch silicon wafer with a backside kept at 20 °C and with aluminium paste to lower the heat. However, fused silica does not have good thermal conductivity to convey the generated heat from the mask surface to the carrier wafer. Meanwhile, the etching process was separated into small intervals to reduce the temperature accumulation. The sample was taken out from the process chamber every 30 min, and was cooled down to room temperature.

**Figure 3:**
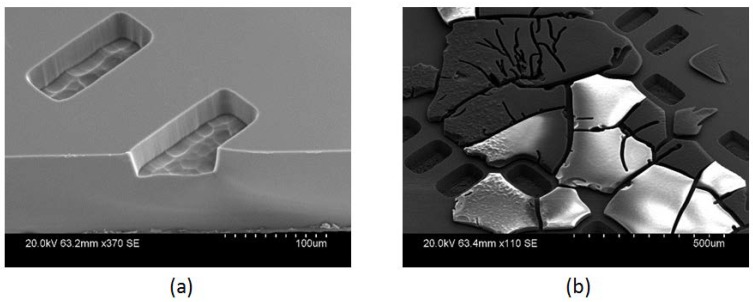
Scanning electron microscopy (SEM) micrographs: (**a**) The etched fused silica using the optimal recipe; (**b**) Over burnt KMPR 1035 residual.

Figure 3 illustrates the scanning electron microscopy (SEM) micrograph of the etched results. The etched via holes had dimensions of 100 μm × 50 μm. Dry etching of the fused silica wafer with ICP RIE gave a smooth sidewall finish and the sidewall angle was above 85°, as shown in Figure 3a. One of the shortcomings of using thick photoresist as the etching mask is illustrated in Figure 3b. The etched sample was cleaned by piranha solution for half an hour, but the burnt residual on top of the fused silica sample cannot be removed.

Another disadvantage of the KMPR 1035 mask is the high stress. Typically, wafers with a thickness over 200 μm do not show any shape change after applying the thick photoresist. However, for an ultra-thin wafer with a thickness of 100 μm, when the photoresist has an average thickness of 70 μm and when it is cooled down to room temperature post-baking, the compressive stress will bend the two-inch fused silica wafer. The etching selectivity using amorphous silicon mask is tripled compared to a single photoresist mask. However, a lot of bubbles appear when the deposition thickness exceeds 6 μm with plasma-enhanced chemical vapor deposition (PECVD). In consequence, a-Si mask is only suitable for via hole etching with a depth less than 25 μm. The chromium mask showed the best selectivity result, and it is possible to etch through 100 μm thick fused silica, if a 5 μm thick chromium mask can be deposited. However, due to the equipment limitations in our lab, the chromium mask was only sputtered for two hours in our experiment, achieving a thickness of 1.4 μm, which is not sufficient to etch through the fused silica. Furthermore, wet etching was used when transferring the desired pattern to the chromium mask, so the isotropic properties will lead to a slightly larger via hole size, which could be a potential drawback.

### 3.2. Laser Ablation of Fused Silica

**Figure 4:**
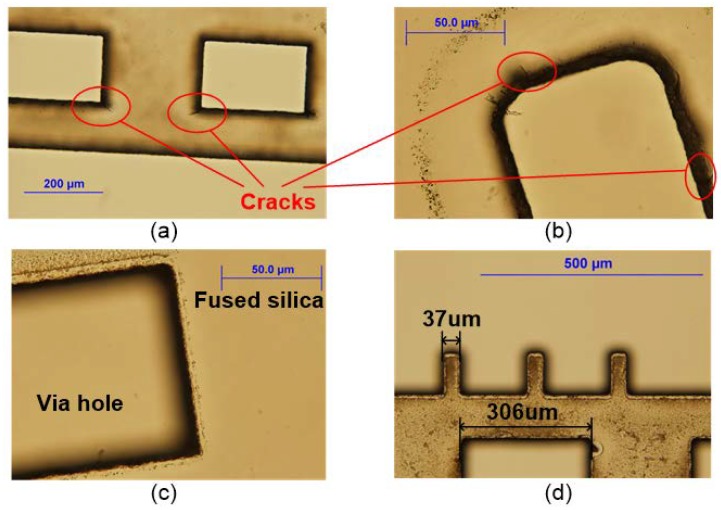
Appearances of etched samples under microscope: (**a**) 115 mW and 50 passes; (**b**) 115 mW and 50 passes with round corners; (**c**) 102 mW and 60 passes; (**d**) Accuracy of laser etching.

It was found empirically that the laser average power and the repetition used for each pattern play key roles in the process. Based on the appearance of the etched sample, the key parameters of the laser ablation recipe were tested and optimized. Figure 4 shows the inspection results under optical microscope, without cleaning the debris.

In the initial experiment, the laser power was set to 70 mW and the repetition was 50 passes. However, it only left faint marks on the wafer without any hole or trench, which means the power was below the ablation threshold. Figure 4a shows the etched result when the power is increased to 115 mW with a repetition of 50 passes. The corner chipped obviously although the central rectangular window fell away from the wafer. Since the chipping positions happened at the four corners, one possible reason could be the sharp angle of the laser scanning route. In order to avoid the abrupt direction change, patterns with round corners were etched with the same processing parameters and the results in Figure 4b show that the cracks still exist at random locations. The other explanation for the cracks is that the laser power was too high for the ultra-thin wafer. The repetition of the etching pattern should be increased, with a weaker average power selected. When processing with 102 mW of power at 60 repetition passes, the fused silica wafer was able to be cleanly etched through without cracks, as demonstrated in Figure 4c. This corresponds to a processing fluence of 2.89 J/cm^2^, while the pulse overlap was 91%. Furthermore, based on these optimized parameters, the power of the initial 10 passes was adjusted to a more gentle 80 mW, but the etched results showed no obvious improvement. This confirms that the roughness of the etched edges and sidewalls is independent of the average output power when it is within a reasonable range. In order to study the etching accuracy and resolution of the laser ablation process, a pattern with tooth structures and open windows was etched and is illustrated in Figure 4d. The width of the tooth part was set to 50 μm, decreasing to 37 μm and the 300 μm wide open windows became 306 μm.

**Figure 5:**
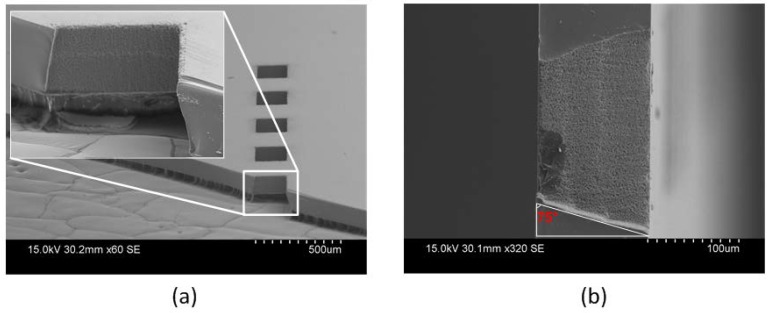
(**a**) SEM micrograph of laser etched via holes; (**b**) Cross-section image, etching direction from right to left.

Subsequently to the laser ablation process, an ultrasonic acetone bath was adopted in order to lift the debris from the surface. Figure 5a shows the SEM micrograph of the through via holes etched by laser. The via holes had dimensions of 200 μm × 300 μm and the distance between the adjacent rectangular windows was 250 μm. The processed surface was free of debris after cleaning and the sidewall has a reasonable roughness to support metal connections. A cross-section of the sidewall profile is illustrated in Figure 5b and the tapered angle was measured to be 75°, which is more suitable for the subsequent metallization process compared to a vertical sidewall. 

**Figure 6:**
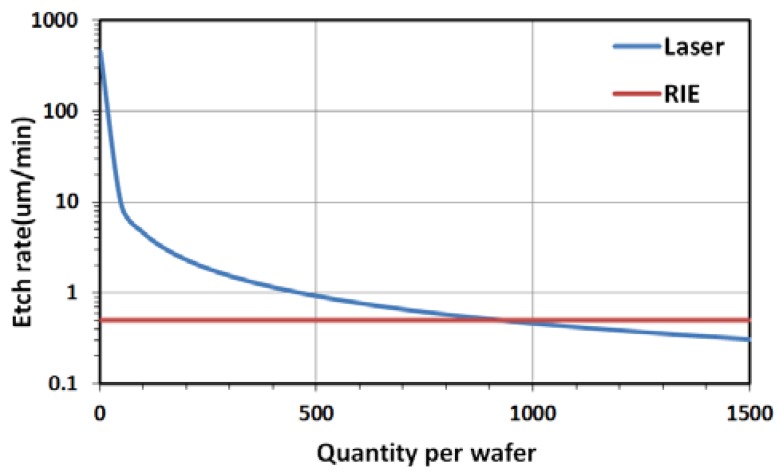
Etch rate comparison.

In order to compare the etch rates between ICP-RIE and laser ablation, a two-inch wafer with 624 via holes was processed by laser machining and the time consumed was 2.3 h, corresponding to 13 s for a single hole etching. The etch rates of both methods are plotted versus via hole numbers in Figure 6. In contrast to laser ablation, which etches the via holes one by one, the etch rate of ICP-RIE will not change with via hole number since they are processed at the same time. The laser ablation method is less time consuming with a via number below 950 per wafer. The comparison results are based on 200 μm × 300 μm via hole dimensions in 100 μm thick, two-inch fused silica wafers. The cross point of the two curves will shift to the right with decreased via hole dimensions, and vice versa.

## 4. Conclusions

In this paper, two technologies—ICP-RIE and laser ablation—which can create through via holes on fused silica wafers were studied and compared. In terms of the dry etching method, we tested three types of mask material and investigated the etch rate and selectivity of each material with the optimal process recipes. Then, we analyzed the advantages and disadvantages of different materials, as well as their ability to etch through 100 μm thick wafers. Experiments show that on the practical semiconductor manufacturing level, chromium metal mask is the best choice for the etching of ultra-thin fused silica wafers. The sidewalls are almost perpendicular to the wafer surface and have a very smooth finish.

We also described the processing strategy and experimental setup when applying a picosecond laser to etch the through via holes. The laser power, kerf size and repetition of the etched pattern are the three main parameters that determine the etched results. Laser ablation is considered a more convenient method to obtain through via holes in thin fused silica wafers, since it can avoid these complex micro-fabrication process steps. The etched via holes show a good quality and sidewall profile for the following metallization process, although the accuracy and surface roughness cannot match the results obtained from dry etching. Considering the fast etch rate when the quantity of holes per wafer is less than 500, this method has extensive potential applications and value.

## Figures and Tables

**Table 1:** Fused silica etch rate and selectivity.

Mask Material	Etch time (min)	Etched SiO_x_ Depth (μm)	Etched Mask Depth (μm)	Etch Rate (μm/min)	Selectivity
KMPR 1035	110	57	43	0.52	1.33
a-Silicon	2.5	1	0.27	0.4	3.7
Chromium	45	22	1	0.49	22
